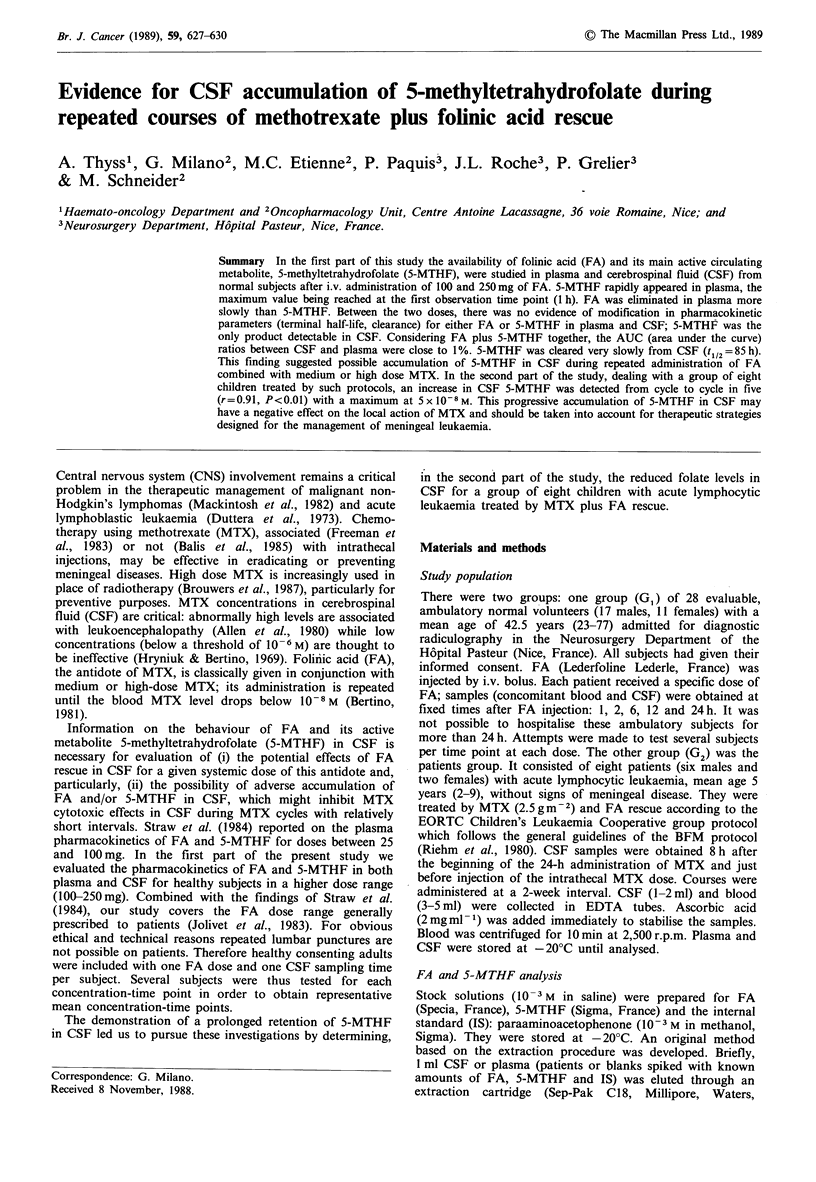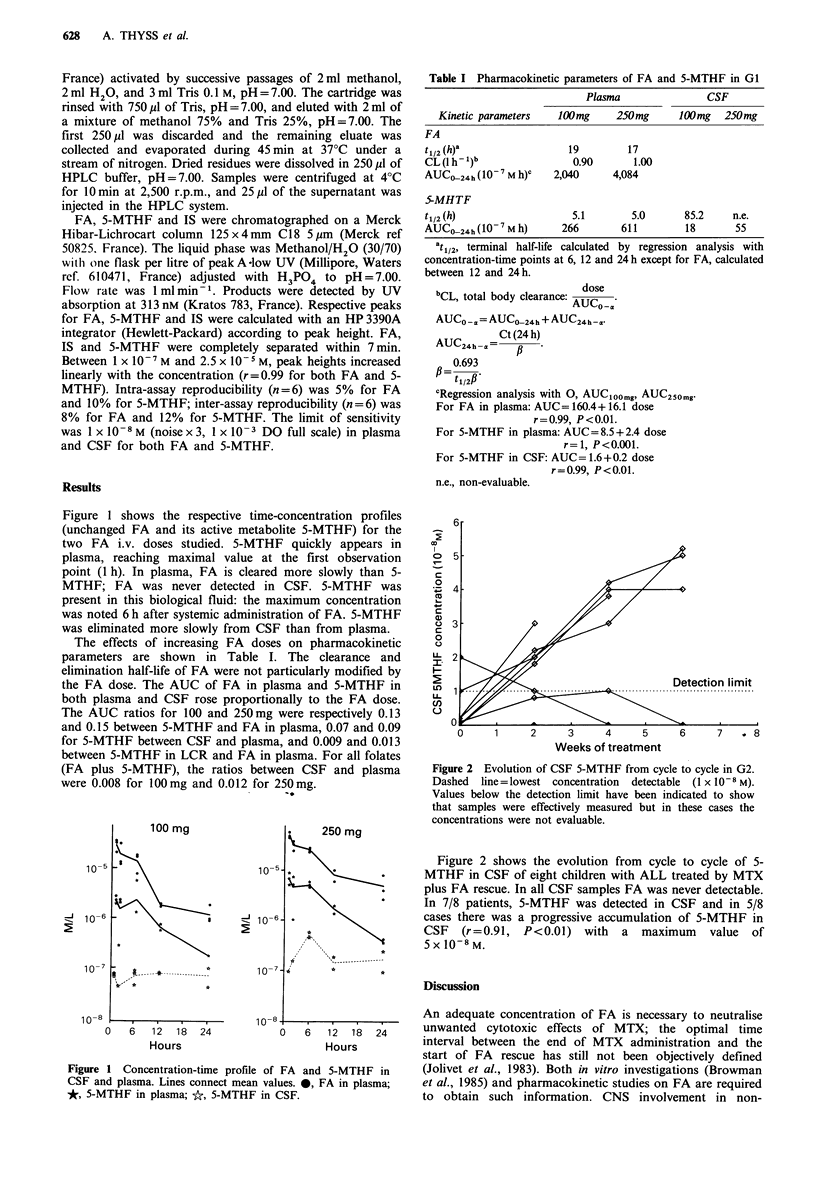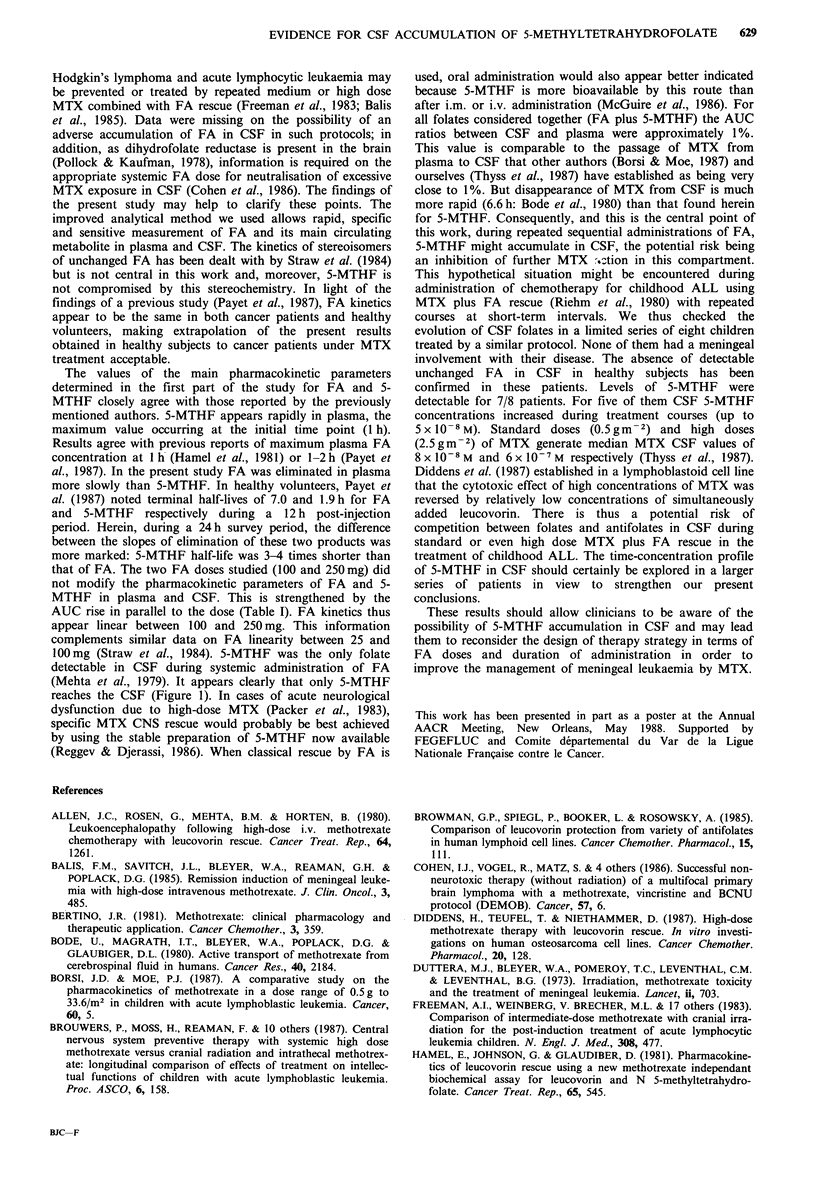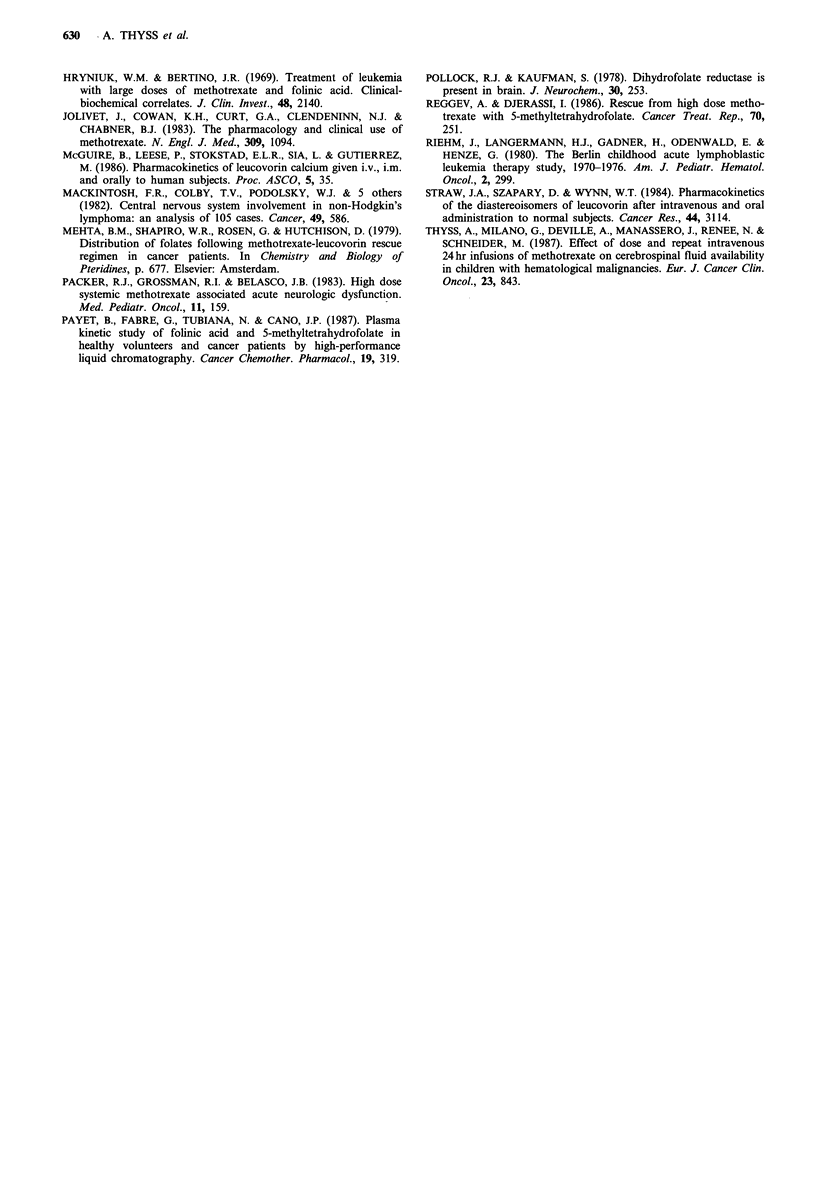# Evidence for CSF accumulation of 5-methyltetrahydrofolate during repeated courses of methotrexate plus folinic acid rescue.

**DOI:** 10.1038/bjc.1989.127

**Published:** 1989-04

**Authors:** A. Thyss, G. Milano, M. C. Etienne, P. Paquis, J. L. Roche, P. Grelier, M. Schneider

**Affiliations:** Haemato-oncology Department, Centre Antoine Lacassagne, Nice, France.

## Abstract

In the first part of this study the availability of folinic acid (FA) and its main active circulating metabolite, 5-methyltetrahydrofolate (5-MTHF), were studied in plasma and cerebrospinal fluid (CSF) from normal subjects after i.v. administration of 100 and 250 mg of FA. 5-MTHF rapidly appeared in plasma, the maximum value being reached at the first observation time point (1 h). FA was eliminated in plasma more slowly than 5-MTHF. Between the two doses, there was no evidence of modification in pharmacokinetic parameters (terminal half-life, clearance) for either FA or 5-MTHF in plasma and CSF; 5-MTHF was the only product detectable in CSF. Considering FA plus 5-MTHF together, the AUC (area under the curve) ratios between CSF and plasma were close to 1%. 5-MTHF was cleared very slowly from CSF (t 1/2 = 85 h). This finding suggested possible accumulation of 5-MTHF in CSF during repeated administration of FA combined with medium or high dose MTX. In the second part of the study, dealing with a group of eight children treated by such protocols, an increase in CSF 5-MTHF was detected from cycle to cycle in five (r = 0.91, P less than 0.01) with a maximum at 5 x 10(-8) M. This progressive accumulation of 5-MTHF in CSF may have a negative effect on the local action of MTX and should be taken into account for therapeutic strategies designed for the management of meningeal leukaemia.


Br. J. Cancer (1989), 59, 627–630 © The Macmillan Press Ltd., 1989

### Evidence for CSF accumulation of 5-methyltetrahydrofolate during repeated courses of methotrexate plus folinic acid rescue

A. Thyss[1], G. Milano[2], M.C. Etienne[2], P. Paquis[3], J.L. Roche[3], P. Grelier[3] & M. Schneider[2]

[1]*Haemato-oncology Department and* [2]*Oncopharmacology Unit, Centre Antoine Lacassagne, 36 voie Romaine, Nice; and* [3]*Neurosurgery Department, Hôpital Pasteur, Nice, France.*

**Summary** In the first part of this study the availability of folinic acid (FA) and its main active circulating metabolite, 5-methyltetrahydrofolate (5-MTHF), were studied in plasma and cerebrospinal fluid (CSF) from normal subjects after i.v. administration of 100 and 250 mg of FA. 5-MTHF rapidly appeared in plasma, the maximum value being reached at the first observation time point (1 h). FA was eliminated in plasma more slowly than 5-MTHF. Between the two doses, there was no evidence of modification in pharmacokinetic parameters (terminal half-life, clearance) for either FA or 5-MTHF in plasma and CSF; 5-MTHF was the only product detectable in CSF. Considering FA plus 5-MTHF together, the AUC (area under the curve) ratios between CSF and plasma were close to 1%. 5-MTHF was cleared very slowly from CSF ($t_{1/2}=85$ h). This finding suggested possible accumulation of 5-MTHF in CSF during repeated administration of FA combined with medium or high dose MTX. In the second part of the study, dealing with a group of eight children treated by such protocols, an increase in CSF 5-MTHF was detected from cycle to cycle in five ($r=0.91$, $P<0.01$) with a maximum at $5\times10^{-8}$ M. This progressive accumulation of 5-MTHF in CSF may have a negative effect on the local action of MTX and should be taken into account for therapeutic strategies designed for the management of meningeal leukaemia.

Central nervous system (CNS) involvement remains a critical problem in the therapeutic management of malignant non-Hodgkin's lymphomas (Mackintosh *et al.*, 1982) and acute lymphoblastic leukaemia (Duttera *et al.*, 1973). Chemotherapy using methotrexate (MTX), associated (Freeman *et al.*, 1983) or not (Balis *et al.*, 1985) with intrathecal injections, may be effective in eradicating or preventing meningeal diseases. High dose MTX is increasingly used in place of radiotherapy (Brouwers *et al.*, 1987), particularly for preventive purposes. MTX concentrations in cerebrospinal fluid (CSF) are critical: abnormally high levels are associated with leukoencephalopathy (Allen *et al.*, 1980) while low concentrations (below a threshold of $10^{-6}$ M) are thought to be ineffective (Hryniuk & Bertino, 1969). Folinic acid (FA), the antidote of MTX, is classically given in conjunction with medium or high-dose MTX; its administration is repeated until the blood MTX level drops below $10^{-8}$ M (Bertino, 1981).

Information on the behaviour of FA and its active metabolite 5-methyltetrahydrofolate (5-MTHF) in CSF is necessary for evaluation of (i) the potential effects of FA rescue in CSF for a given systemic dose of this antidote and, particularly, (ii) the possibility of adverse accumulation of FA and/or 5-MTHF in CSF, which might inhibit MTX cytotoxic effects in CSF during MTX cycles with relatively short intervals. Straw *et al.* (1984) reported on the plasma pharmacokinetics of FA and 5-MTHF for doses between 25 and 100 mg. In the first part of the present study we evaluated the pharmacokinetics of FA and 5-MTHF in both plasma and CSF for healthy subjects in a higher dose range (100–250 mg). Combined with the findings of Straw *et al.* (1984), our study covers the FA dose range generally prescribed to patients (Jolivet *et al.*, 1983). For obvious ethical and technical reasons repeated lumbar punctures are not possible on patients. Therefore healthy consenting adults were included with one FA dose and one CSF sampling time per subject. Several subjects were thus tested for each concentration-time point in order to obtain representative mean concentration-time points.

The demonstration of a prolonged retention of 5-MTHF in CSF led us to pursue these investigations by determining, in the second part of the study, the reduced folate levels in CSF for a group of eight children with acute lymphocytic leukaemia treated by MTX plus FA rescue.

Correspondence: G. Milano.
Received 8 November, 1988.

### Materials and methods

### *Study population*

There were two groups: one group ($G_1$) of 28 evaluable, ambulatory normal volunteers (17 males, 11 females) with a mean age of 42.5 years (23–77) admitted for diagnostic radiculography in the Neurosurgery Department of the Hôpital Pasteur (Nice, France). All subjects had given their informed consent. FA (Lederfoline Lederle, France) was injected by i.v. bolus. Each patient received a specific dose of FA; samples (concomitant blood and CSF) were obtained at fixed times after FA injection: 1, 2, 6, 12 and 24 h. It was not possible to hospitalise these ambulatory subjects for more than 24 h. Attempts were made to test several subjects per time point at each dose. The other group ($G_2$) was the patients group. It consisted of eight patients (six males and two females) with acute lymphocytic leukaemia, mean age 5 years (2–9), without signs of meningeal disease. They were treated by MTX (2.5 g m$^{-2}$) and FA rescue according to the EORTC Children's Leukaemia Cooperative group protocol which follows the general guidelines of the BFM protocol (Riehm *et al.*, 1980). CSF samples were obtained 8 h after the beginning of the 24-h administration of MTX and just before injection of the intrathecal MTX dose. Courses were administered at a 2-week interval. CSF (1–2 ml) and blood (3–5 ml) were collected in EDTA tubes. Ascorbic acid (2 mg ml$^{-1}$) was added immediately to stabilise the samples. Blood was centrifuged for 10 min at 2,500 r.p.m. Plasma and CSF were stored at $-20$°C until analysed.

### *FA and 5-MTHF analysis*

Stock solutions ($10^{-3}$ M in saline) were prepared for FA (Specia, France), 5-MTHF (Sigma, France) and the internal standard (IS): paraaminoacetophenone ($10^{-3}$ M in methanol, Sigma). They were stored at $-20$°C. An original method based on the extraction procedure was developed. Briefly, 1 ml CSF or plasma (patients or blanks spiked with known amounts of FA, 5-MTHF and IS) was eluted through an extraction cartridge (Sep-Pak C18, Millipore, Waters,

France) activated by successive passages of 2 ml methanol, 2 ml $H_2O$, and 3 ml Tris 0.1 M, pH = 7.00. The cartridge was rinsed with 750 $\mu$l of Tris, pH = 7.00, and eluted with 2 ml of a mixture of methanol 75% and Tris 25%, pH = 7.00. The first 250 $\mu$l was discarded and the remaining eluate was collected and evaporated during 45 min at 37°C under a stream of nitrogen. Dried residues were dissolved in 250 $\mu$l of HPLC buffer, pH = 7.00. Samples were centrifuged at 4°C for 10 min at 2,500 r.p.m., and 25 $\mu$l of the supernatant was injected in the HPLC system.

FA, 5-MTHF and IS were chromatographed on a Merck Hibar-Lichrocart column 125 × 4 mm C18 5 $\mu$m (Merck ref 50825, France). The liquid phase was Methanol/$H_2O$ (30/70) with one flask per litre of peak A-low UV (Millipore, Waters ref. 610471, France) adjusted with $H_3PO_4$ to pH = 7.00. Flow rate was 1 ml $min^{-1}$. Products were detected by UV absorption at 313 nM (Kratos 783, France). Respective peaks for FA, 5-MTHF and IS were calculated with an HP 3390A integrator (Hewlett-Packard) according to peak height. FA, IS and 5-MTHF were completely separated within 7 min. Between $1 \times 10^{-7}$ M and $2.5 \times 10^{-5}$ M, peak heights increased linearly with the concentration ($r = 0.99$ for both FA and 5-MTHF). Intra-assay reproducibility ($n = 6$) was 5% for FA and 10% for 5-MTHF; inter-assay reproducibility ($n = 6$) was 8% for FA and 12% for 5-MTHF. The limit of sensitivity was $1 \times 10^{-8}$ M (noise × 3, $1 \times 10^{-3}$ DO full scale) in plasma and CSF for both FA and 5-MTHF.

### Results

Figure 1 shows the respective time-concentration profiles (unchanged FA and its active metabolite 5-MTHF) for the two FA i.v. doses studied. 5-MTHF quickly appears in plasma, reaching maximal value at the first observation point (1 h). In plasma, FA is cleared more slowly than 5-MTHF; FA was never detected in CSF. 5-MTHF was present in this biological fluid: the maximum concentration was noted 6 h after systemic administration of FA. 5-MTHF was eliminated more slowly from CSF than from plasma.

The effects of increasing FA doses on pharmacokinetic parameters are shown in Table I. The clearance and elimination half-life of FA were not particularly modified by the FA dose. The AUC of FA in plasma and 5-MTHF in both plasma and CSF rose proportionally to the FA dose. The AUC ratios for 100 and 250 mg were respectively 0.13 and 0.15 between 5-MTHF and FA in plasma, 0.07 and 0.09 for 5-MTHF between CSF and plasma, and 0.009 and 0.013 between 5-MTHF in LCR and FA in plasma. For all folates (FA plus 5-MTHF), the ratios between CSF and plasma were 0.008 for 100 mg and 0.012 for 250 mg.

**Figure 1** Concentration-time profile of FA and 5-MTHF in CSF and plasma. Lines connect mean values. ●, FA in plasma; ★, 5-MTHF in plasma; ☆, 5-MTHF in CSF.

**Table I** Pharmacokinetic parameters of FA and 5-MTHF in G1

| | *Plasma* | | *CSF* | |
|---|---|---|---|---|
| *Kinetic parameters* | *100 mg* | *250 mg* | *100 mg* | *250 mg* |
| *FA* | | | | |
| $t_{1/2}$ (h)[a] | 19 | 17 | | |
| CL ($l\,h^{-1}$)[b] | 0.90 | 1.00 | | |
| $AUC_{0-24h}$ ($10^{-7}$ M h)[c] | 2,040 | 4,084 | | |
| *5-MHTF* | | | | |
| $t_{1/2}$ (h) | 5.1 | 5.0 | 85.2 | n.e. |
| $AUC_{0-24h}$ ($10^{-7}$ M h) | 266 | 611 | 18 | 55 |

[a]$t_{1/2}$, terminal half-life calculated by regression analysis with concentration-time points at 6, 12 and 24 h except for FA, calculated between 12 and 24 h.

[b]CL, total body clearance: $\frac{\text{dose}}{AUC_{0-\alpha}}$.

$AUC_{0-\alpha} = AUC_{0-24h} + AUC_{24h-\alpha}$.

$AUC_{24h-\alpha} = \frac{Ct(24\,h)}{\beta}$.

$\beta = \frac{0.693}{t_{1/2}\beta}$.

[c]Regression analysis with O, $AUC_{100mg}$, $AUC_{250mg}$.
For FA in plasma: AUC = 160.4 + 16.1 dose
$r = 0.99$, $P < 0.01$.
For 5-MTHF in plasma: AUC = 8.5 + 2.4 dose
$r = 1$, $P < 0.001$.
For 5-MTHF in CSF: AUC = 1.6 + 0.2 dose
$r = 0.99$, $P < 0.01$.
n.e., non-evaluable.

**Figure 2** Evolution of CSF 5-MTHF from cycle to cycle in G2. Dashed line = lowest concentration detectable ($1 \times 10^{-8}$ M). Values below the detection limit have been indicated to show that samples were effectively measured but in these cases the concentrations were not evaluable.

Figure 2 shows the evolution from cycle to cycle of 5-MTHF in CSF of eight children with ALL treated by MTX plus FA rescue. In all CSF samples FA was never detectable. In 7/8 patients, 5-MTHF was detected in CSF and in 5/8 cases there was a progressive accumulation of 5-MTHF in CSF ($r = 0.91$, $P < 0.01$) with a maximum value of $5 \times 10^{-8}$ M.

### Discussion

An adequate concentration of FA is necessary to neutralise unwanted cytotoxic effects of MTX; the optimal time interval between the end of MTX administration and the start of FA rescue has still not been objectively defined (Jolivet et al., 1983). Both in vitro investigations (Browman et al., 1985) and pharmacokinetic studies on FA are required to obtain such information. CNS involvement in non-

Hodgkin's lymphoma and acute lymphocytic leukaemia may be prevented or treated by repeated medium or high dose MTX combined with FA rescue (Freeman *et al.*, 1983; Balis *et al.*, 1985). Data were missing on the possibility of an adverse accumulation of FA in CSF in such protocols; in addition, as dihydrofolate reductase is present in the brain (Pollock & Kaufman, 1978), information is required on the appropriate systemic FA dose for neutralisation of excessive MTX exposure in CSF (Cohen *et al.*, 1986). The findings of the present study may help to clarify these points. The improved analytical method we used allows rapid, specific and sensitive measurement of FA and its main circulating metabolite in plasma and CSF. The kinetics of stereoisomers of unchanged FA has been dealt with by Straw *et al.* (1984) but is not central in this work and, moreover, 5-MTHF is not compromised by this stereochemistry. In light of the findings of a previous study (Payet *et al.*, 1987), FA kinetics appear to be the same in both cancer patients and healthy volunteers, making extrapolation of the present results obtained in healthy subjects to cancer patients under MTX treatment acceptable.

The values of the main pharmacokinetic parameters determined in the first part of the study for FA and 5-MTHF closely agree with those reported by the previously mentioned authors. 5-MTHF appears rapidly in plasma, the maximum value occurring at the initial time point (1 h). Results agree with previous reports of maximum plasma FA concentration at 1 h (Hamel *et al.*, 1981) or 1–2 h (Payet *et al.*, 1987). In the present study FA was eliminated in plasma more slowly than 5-MTHF. In healthy volunteers, Payet *et al.* (1987) noted terminal half-lives of 7.0 and 1.9 h for FA and 5-MTHF respectively during a 12 h post-injection period. Herein, during a 24 h survey period, the difference between the slopes of elimination of these two products was more marked: 5-MTHF half-life was 3–4 times shorter than that of FA. The two FA doses studied (100 and 250 mg) did not modify the pharmacokinetic parameters of FA and 5-MTHF in plasma and CSF. This is strengthened by the AUC rise in parallel to the dose (Table I). FA kinetics thus appear linear between 100 and 250 mg. This information complements similar data on FA linearity between 25 and 100 mg (Straw *et al.*, 1984). 5-MTHF was the only folate detectable in CSF during systemic administration of FA (Mehta *et al.*, 1979). It appears clearly that only 5-MTHF reaches the CSF (Figure 1). In cases of acute neurological dysfunction due to high-dose MTX (Packer *et al.*, 1983), specific MTX CNS rescue would probably be best achieved by using the stable preparation of 5-MTHF now available (Reggev & Djerassi, 1986). When classical rescue by FA is used, oral administration would also appear better indicated because 5-MTHF is more bioavailable by this route than after i.m. or i.v. administration (McGuire *et al.*, 1986). For all folates considered together (FA plus 5-MTHF) the AUC ratios between CSF and plasma were approximately 1%. This value is comparable to the passage of MTX from plasma to CSF that other authors (Borsi & Moe, 1987) and ourselves (Thyss *et al.*, 1987) have established as being very close to 1%. But disappearance of MTX from CSF is much more rapid (6.6 h: Bode *et al.*, 1980) than that found herein for 5-MTHF. Consequently, and this is the central point of this work, during repeated sequential administrations of FA, 5-MTHF might accumulate in CSF, the potential risk being an inhibition of further MTX action in this compartment. This hypothetical situation might be encountered during administration of chemotherapy for childhood ALL using MTX plus FA rescue (Riehm *et al.*, 1980) with repeated courses at short-term intervals. We thus checked the evolution of CSF folates in a limited series of eight children treated by a similar protocol. None of them had a meningeal involvement with their disease. The absence of detectable unchanged FA in CSF in healthy subjects has been confirmed in these patients. Levels of 5-MTHF were detectable for 7/8 patients. For five of them CSF 5-MTHF concentrations increased during treatment courses (up to $5 \times 10^{-8}$ M). Standard doses ($0.5\,g\,m^{-2}$) and high doses ($2.5\,g\,m^{-2}$) of MTX generate median MTX CSF values of $8 \times 10^{-8}$ M and $6 \times 10^{-7}$ M respectively (Thyss *et al.*, 1987). Diddens *et al.* (1987) established in a lymphoblastoid cell line that the cytotoxic effect of high concentrations of MTX was reversed by relatively low concentrations of simultaneously added leucovorin. There is thus a potential risk of competition between folates and antifolates in CSF during standard or even high dose MTX plus FA rescue in the treatment of childhood ALL. The time-concentration profile of 5-MTHF in CSF should certainly be explored in a larger series of patients in view to strengthen our present conclusions.

These results should allow clinicians to be aware of the possibility of 5-MTHF accumulation in CSF and may lead them to reconsider the design of therapy strategy in terms of FA doses and duration of administration in order to improve the management of meningeal leukaemia by MTX.

This work has been presented in part as a poster at the Annual AACR Meeting, New Orleans, May 1988. Supported by FEGEFLUC and Comite départemental du Var de la Ligue Nationale Française contre le Cancer.

### References

ALLEN, J.C., ROSEN, G., MEHTA, B.M. & HORTEN, B. (1980). Leukoencephalopathy following high-dose i.v. methotrexate chemotherapy with leucovorin rescue. *Cancer Treat. Rep.*, **64**, 1261.

BALIS, F.M., SAVITCH, J.L., BLEYER, W.A., REAMAN, G.H. & POPLACK, D.G. (1985). Remission induction of meningeal leukemia with high-dose intravenous methotrexate. *J. Clin. Oncol.*, **3**, 485.

BERTINO, J.R. (1981). Methotrexate: clinical pharmacology and therapeutic application. *Cancer Chemother.*, **3**, 359.

BODE, U., MAGRATH, I.T., BLEYER, W.A., POPLACK, D.G. & GLAUBIGER, D.L. (1980). Active transport of methotrexate from cerebrospinal fluid in humans. *Cancer Res.*, **40**, 2184.

BORSI, J.D. & MOE, P.J. (1987). A comparative study on the pharmacokinetics of methotrexate in a dose range of 0.5 g to 33.6/m² in children with acute lymphoblastic leukemia. *Cancer*, **60**, 5.

BROUWERS, P., MOSS, H., REAMAN, F. & 10 others (1987). Central nervous system preventive therapy with systemic high dose methotrexate versus cranial radiation and intrathecal methotrexate: longitudinal comparison of effects of treatment on intellectual functions of children with acute lymphoblastic leukemia. *Proc. ASCO*, **6**, 158.

BROWMAN, G.P., SPIEGL, P., BOOKER, L. & ROSOWSKY, A. (1985). Comparison of leucovorin protection from variety of antifolates in human lymphoid cell lines. *Cancer Chemother. Pharmacol.*, **15**, 111.

COHEN, I.J., VOGEL, R., MATZ, S. & 4 others (1986). Successful non-neurotoxic therapy (without radiation) of a multifocal primary brain lymphoma with a methotrexate, vincristine and BCNU protocol (DEMOB). *Cancer*, **57**, 6.

DIDDENS, H., TEUFEL, T. & NIETHAMMER, D. (1987). High-dose methotrexate therapy with leucovorin rescue. *In vitro* investigations on human osteosarcoma cell lines. *Cancer Chemother. Pharmacol.*, **20**, 128.

DUTTERA, M.J., BLEYER, W.A., POMEROY, T.C., LEVENTHAL, C.M. & LEVENTHAL, B.G. (1973). Irradiation, methotrexate toxicity and the treatment of meningeal leukemia. *Lancet*, **ii**, 703.

FREEMAN, A.I., WEINBERG, V. BRECHER, M.L. & 17 others (1983). Comparison of intermediate-dose methotrexate with cranial irradiation for the post-induction treatment of acute lymphocytic leukemia children. *N. Engl. J. Med.*, **308**, 477.

HAMEL, E., JOHNSON, G. & GLAUDIBER, D. (1981). Pharmacokinetics of leucovorin rescue using a new methotrexate independant biochemical assay for leucovorin and N 5-methyltetrahydrofolate. *Cancer Treat. Rep.*, **65**, 545.

HRYNIUK, W.M. & BERTINO, J.R. (1969). Treatment of leukemia with large doses of methotrexate and folinic acid. Clinical-biochemical correlates. *J. Clin. Invest.*, **48**, 2140.

JOLIVET, J., COWAN, K.H., CURT, G.A., CLENDENINN, N.J. & CHABNER, B.J. (1983). The pharmacology and clinical use of methotrexate. *N. Engl. J. Med.*, **309**, 1094.

McGUIRE, B., LEESE, P., STOKSTAD, E.L.R., SIA, L. & GUTIERREZ, M. (1986). Pharmacokinetics of leucovorin calcium given i.v., i.m. and orally to human subjects. *Proc. ASCO*, **5**, 35.

MACKINTOSH, F.R., COLBY, T.V., PODOLSKY, W.J. & 5 others (1982). Central nervous system involvement in non-Hodgkin's lymphoma: an analysis of 105 cases. *Cancer*, **49**, 586.

MEHTA, B.M., SHAPIRO, W.R., ROSEN, G. & HUTCHISON, D. (1979). Distribution of folates following methotrexate-leucovorin rescue regimen in cancer patients. In *Chemistry and Biology of Pteridines*, p. 677. Elsevier: Amsterdam.

PACKER, R.J., GROSSMAN, R.I. & BELASCO, J.B. (1983). High dose systemic methotrexate associated acute neurologic dysfunction. *Med. Pediatr. Oncol.*, **11**, 159.

PAYET, B., FABRE, G., TUBIANA, N. & CANO, J.P. (1987). Plasma kinetic study of folinic acid and 5-methyltetrahydrofolate in healthy volunteers and cancer patients by high-performance liquid chromatography. *Cancer Chemother. Pharmacol.*, **19**, 319.

POLLOCK, R.J. & KAUFMAN, S. (1978). Dihydrofolate reductase is present in brain. *J. Neurochem.*, **30**, 253.

REGGEV, A. & DJERASSI, I. (1986). Rescue from high dose methotrexate with 5-methyltetrahydrofolate. *Cancer Treat. Rep.*, **70**, 251.

RIEHM, J., LANGERMANN, H.J., GADNER, H., ODENWALD, E. & HENZE, G. (1980). The Berlin childhood acute lymphoblastic leukemia therapy study, 1970–1976. *Am. J. Pediatr. Hematol. Oncol.*, **2**, 299.

STRAW, J.A., SZAPARY, D. & WYNN, W.T. (1984). Pharmacokinetics of the diastereoisomers of leucovorin after intravenous and oral administration to normal subjects. *Cancer Res.*, **44**, 3114.

THYSS, A., MILANO, G., DEVILLE, A., MANASSERO, J., RENEE, N. & SCHNEIDER, M. (1987). Effect of dose and repeat intravenous 24 hr infusions of methotrexate on cerebrospinal fluid availability in children with hematological malignancies. *Eur. J. Cancer Clin. Oncol.*, **23**, 843.